# Expression of the Hippo transducer TAZ in association with WNT pathway mutations impacts survival outcomes in advanced gastric cancer patients treated with first-line chemotherapy

**DOI:** 10.1186/s12967-018-1385-y

**Published:** 2018-02-05

**Authors:** Elisa Melucci, Beatrice Casini, Livia Ronchetti, Laura Pizzuti, Francesca Sperati, Matteo Pallocca, Francesca De Nicola, Frauke Goeman, Enzo Gallo, Carla Azzurra Amoreo, Domenico Sergi, Irene Terrenato, Patrizia Vici, Luigi Di Lauro, Maria Grazia Diodoro, Edoardo Pescarmona, Maddalena Barba, Marco Mazzotta, Marcella Mottolese, Maurizio Fanciulli, Gennaro Ciliberto, Ruggero De Maria, Simonetta Buglioni, Marcello Maugeri-Saccà

**Affiliations:** 1 Department of Pathology, “Regina Elena” National Cancer Institute, Via Elio Chianesi 53, 00144 Rome, Italy; 2 Division of Medical Oncology 2, “Regina Elena” National Cancer Institute, Via Elio Chianesi 53, 00144 Rome, Italy; 3 Biostatistics-Scientific Direction, “Regina Elena” National Cancer Institute, Via Elio Chianesi 53, 00144 Rome, Italy; 4 SAFU Laboratory, Department of Research, Advanced Diagnostic, and Technological Innovation, “Regina Elena” National Cancer Institute, Via Elio Chianesi 53, 00144 Rome, Italy; 5 Oncogenomic and Epigenetic Unit, “Regina Elena” National Cancer Institute, Via Elio Chianesi 53, 00144 Rome, Italy; 6 Scientific Direction, “Regina Elena” National Cancer Institute, Via Elio Chianesi 53, 00144 Rome, Italy; 7 Medical Oncology Unit, Policlinico Sant’Andrea, Via Di Grotta Rossa, 1035/1039, 00189 Rome, Italy; 8 Institute of General Pathology, Catholic University of the Sacred Heart, Largo Agostino Gemelli, 10, 00168 Rome, Italy

**Keywords:** Gastric cancer, Hippo pathway, YAP, TAZ, Wnt pathway, CTNNB1, APC, FBXW7

## Abstract

**Background:**

An extensive crosstalk co-regulates the Hippo and Wnt pathway. Preclinical studies revealed that the Hippo transducers YAP/TAZ mediate a number of oncogenic functions in gastric cancer (GC). Moreover, comprehensive characterization of GC demonstrated that the Wnt pathway is targeted by oncogenic mutations. On this ground, we hypothesized that YAP/TAZ- and Wnt-related biomarkers may predict clinical outcomes in GC patients treated with chemotherapy.

**Methods:**

In the present study, we included 86 patients with advanced GC treated with first-line chemotherapy in prospective phase II trials or in routine clinical practice. Tissue samples were immunostained to evaluate the expression of YAP/TAZ. Mutational status of key Wnt pathway genes (*CTNNB1*, *APC* and *FBXW7*) was assessed by targeted DNA next-generation sequencing (NGS). Survival curves were estimated and compared by the Kaplan–Meier product-limit method and the log-rank test, respectively. Variables potentially affecting progression-free survival (PFS) were verified in univariate Cox proportional hazard models. The final multivariate Cox models were obtained with variables testing significant at the univariate analysis, and by adjusting for all plausible predictors of the outcome of interest (PFS).

**Results:**

We observed a significant association between TAZ expression and Wnt mutations (Chi-squared p = 0.008). Combined TAZ expression and Wnt mutations (TAZ^pos^/WNT^mut^) was more frequently observed in patients with the shortest progression-free survival (negative outliers) (Fisher p = 0.021). Uni-and multivariate Cox regression analyses revealed that patients whose tumors harbored the TAZ^pos^/WNT^mut^ signature had an increased risk of disease progression (univariate Cox: HR 2.27, 95% CI 1.27–4.05, p = 0.006; multivariate Cox: HR 2.73, 95% CI 1.41–5.29, p = 0.003). Finally, the TAZ^pos^/WNT^mut^ signature negatively impacted overall survival.

**Conclusions:**

Collectively, our findings indicate that the oncogenic YAP/TAZ–Wnt crosstalk may be active in GC, conferring chemoresistant traits that translate into adverse survival outcomes.

**Electronic supplementary material:**

The online version of this article (10.1186/s12967-018-1385-y) contains supplementary material, which is available to authorized users.

## Background

Over the past two decades, a wave of studies in flies elucidated the central role of the Hippo pathway in organ development [1]. Ablation of a set of genes including *Warts* (*wts*), *Hippo* (*hpo*), *Salvador* (*sav*) and *Mob as tumor suppressor* (*mats*) led to a remarkable tissue overgrowth, a process tied to increased cellular proliferation and reduced apoptosis [2–11]. These alterations were phenocopied upon the forced over-expression of the transcriptional co-activator *Yorkie* (*yki*) [12]. Complemented by functional and biochemical evidence, studies in *Drosophila* deciphered the functional architecture of the “Salvador–Warts–Hippo” (SWH) pathway, and have been instrumental for characterizing the Hippo pathway in mammals. Indeed, the use of conditional knockout alleles and inducible transgenic mice revealed that manipulation of Hippo pathway components resulted in tissue overgrowth and tumorigenesis [13, 14]. Functionally, Hippo is organized into a core regulatory module and a transcriptional module. The first is composed by the kinases sterile 20-like kinase 1 and 2 (MST1 and MST2; Hpo in *Drosophila*) and large tumor suppressor 1 and 2 (LATS1 and LATS2, Wts in *Drosophila*), together with the adaptor proteins Salvador homolog 1 (SAV1; Sav in *Drosophila*) and MOB kinase activator 1A and 1B (MOB1A and MOB1B; Mats in *Drosophila*). The latter encompasses the transcriptional cofactors yes-associated protein and its paralog transcriptional co-activator with PDZ-binding motif (YAP and TAZ, respectively; Yki in *Drosophila*), along with their transcriptional partners TEA domain-containing sequence-specific transcription factors (TEAD1-4; Scalloped in *Drosophila*) [1]. The core module orchestrates a phosphorylation cascade that results in the inhibition of YAP/TAZ, promoting their nuclear exclusion, cytoplasmic retention and proteasomal degradation [14–18]. When inactivated, or in the presence of stimuli that bypass its function, YAP/TAZ accumulate into the nucleus, interact with their transcriptional partners and ultimately promote the transcription of target genes. Given that loss-of-function of Hippo kinases and adaptors fuelled tumor formation in animal models, and a similar outcome was observed upon the forced expression of Hippo transducers, Hippo was designated as a tumor suppressive signaling deputed to inhibit the oncogenic proteins YAP and TAZ [1].

Hippo signaling lies at the centerpiece of an intricate molecular network [19, 20]. Indeed, a number of regulatory branches modulate its activity, spanning from cell polarity and cell adhesion factors to kinases acting upstream the regulatory module, mechanical forces (mechanotransduction), G-protein-coupled receptors (GPCRs) and metabolic routes [1]. An emerging level of regulation refers to the cooperation between Hippo and the Wnt pathway [1]. Central in the regulation of the Wnt signaling is the β-catenin destruction complex [21]. This is composed by a set of proteins that, in the absence of Wnt ligand stimulation, retains β-catenin in the cytoplasm and enables its degradation, thus preventing β-catenin nuclear translocation and transcription of target genes [21]. The crosstalk between Hippo and Wnt prevalently takes place at the level of β-catenin regulation [22, 23]. Two not mutually exclusive models have been proposed that functionally concatenate these two pathways. The first envisions the incorporation of YAP/TAZ in the β-catenin destruction complex [22]. When the Wnt pathway is in the off state, YAP/TAZ participate in β-catenin degradation, whereas stimulation by Wnt ligands disassembles the complex promoting nuclear accumulation of both YAP/TAZ and β-catenin [22]. The second model proposes that Adenomatosis Polyposis Coli (APC), a central component of the β-catenin destruction complex, serves as a scaffold protein whose correct function is instrumental for the activation of Hippo kinases and consequent inhibition of YAP/TAZ [23]. Consistently, loss of APC disables Hippo-mediated control of YAP/TAZ [23].

Functional in vitro and in vivo studies linked aberrant activation of YAP/TAZ to the progression of gastric cancer (GC) [24], and the inhibition of the YAP/TAZ–TEAD interaction achieved with a Vgl-like-4—(VGLL4) mimicking peptide severely impaired GC cell survival [25]. Moreover, the comprehensive characterization of GC carried out by The Cancer Genome Atlas (TCGA) network revealed oncogenic mutations in central Wnt pathway components, including *CTNNB1* (β-catenin), *APC* and *FBXW7* (F-box/WD repeat domain-containing 7), an antagonist of the Wnt signaling that targets β-catenin for degradation [26]. On this ground, we hypothesized that the Hippo–Wnt pathway crosstalk may be active in GC, conferring more aggressive molecular traits that translate into adverse survival outcomes. To test this hypothesis, tissue samples from 86 GC patients treated with first-line chemotherapy, either in prospective phase II trials or in routine clinical practice [27–30], were retrospectively evaluated by immunohistochemistry (IHC) for assessing the expression of YAP and TAZ. Immunohistochemical characterization was integrated with targeted DNA next-generation sequencing (NGS) analysis of *CTNNB1*, *APC* and *FBXW7*.

## Methods

### Patients and treatment

In the present study, we included 86 patients with histologically confirmed, inoperable locally advanced or metastatic cancer of the stomach or gastroesophageal junction who received first-line chemotherapy (August 2001–June 2015). Median follow-up was 11 months (IQR 5.5–20.5 months). Eligibility was defined by the following criteria: (i) available data on clinical features, administered therapies and treatment outcomes, (ii) complete data on protein biomarkers (YAP and TAZ), and (iii) complete data on Wnt pathway component mutations. Chemotherapy regimens and schedules are detailed in Additional file 1. Tumor responses were evaluated by Response Evaluation Criteria in Solid Tumors (RECIST) criteria v.1.1. Progression-free survival (PFS) was calculated as the time between the first cycle of chemotherapy and radiological evidence of disease progression or death due to any cause. Overall survival (OS) was computed as the time from the first cycle of chemotherapy to death from any cause, and as the time from diagnosis to death due to any cause. Written informed consents were obtained by all the participants. The study was conducted in accordance with the Declaration of Helsinki and approved by the Ethics Committee of the “Regina Elena” National Cancer Institute of Rome. This study adheres to the REMARK guidelines [31].

### Immunohistochemical assessment of YAP and TAZ

The immunohistochemical assessment of YAP and TAZ was performed in formalin-fixed paraffin-embedded (FFPE) tissues from biopsies or surgical samples, and was carried out with the following antibodies: anti-YAP monoclonal antibody (MoAb) (H-9, Santa Cruz) at the dilution of 1:200 and anti-TAZ MoAb (M2-616, BD Pharmingen) at the dilution of 1:400. Immunoreactions were revealed by a streptavidin–biotin enhanced immunoperoxidase technique (Super Sensitive MultiLink, Leica, Milan, Italy) in an automated autostainer (Bond III, Leica). YAP/TAZ expression was reported both in terms of percentage of tumor-expressing cells and staining intensity (0 = absent, 1+ = weak, 2+ = moderate, and 3+ = strong). For tumors with both nuclear and cytoplasmic expression, staining intensity and percentage of tumor-expressing cells were independently assessed in, and reported for, the two cellular compartments. Tumors were classified as negative (YAP^neg^, TAZ^neg^) or positive (YAP^pos^, TAZ^pos^) on the basis of cellular localization and percentage of tumor-expressing cells. YAP/TAZ positivity was defined as a distinct nuclear immunoreactivity in ≥ 20% of neoplastic cells, a classification comparable to that of our previous studies [32–34]. Representative examples of immunohistochemical expression of YAP/TAZ is provided in Additional file 2. Immunoreactivity was evaluated by two investigators blinded to treatment outcomes (EM and LR), and discordant cases were reviewed by a third observer (MM).

### Targeted DNA NGS

DNA was extracted from FFPE tumor blocks using the QIAamp DNA Mini Kit (Qiagen). Quantity of the extracted DNA was assessed by the Qubit dsDNA High Sensitivity Assay Kit on Qubit Fluorometer (Thermofisher Scientific). Library preparation was performed on 20 ng DNA by the Ion AmpliSeq Library 96LV Kit 2.0 (Thermofisher Scientific) and the Wnt custom panel (Thermofisher Scientific), which targets 3 genes (*APC*, *CTNNB1* e *FBXW7*) and generates 174 amplicons (mean coverage: 98.8%). Each library was barcoded with the Ion Xpress Barcode Adapters 1–16 Kit (Thermofisher Scientific) and diluted to a final concentration of 100 pM; barcoded libraries were pooled in equimolar amount and diluted to 35 pM for downstream template preparation. Template preparation was performed by the Ion Chef system (Thermofisher Scientific), which integrates library amplification, ISP recovery-enrichment and Chip loading. Sequencing was performed on Ion S5 system (Thermofisher Scientific) with the Ion 520 chip. Raw data were analyzed using the Torrent Suite Software v.5.2 (Thermofisher Scientific). The coverage analysis was performed using the coverage analysis plug-in v5.2.1.2. All cases had a number of mapped reads > 100.000 and/or the average base coverage > 500×. Polymorphic variants were filtered out exploiting the Ion Reporter Suite (Thermofisher Scientific). Nucleotide variants with an allele frequency less than 3% were not considered. All variants were manually reviewed with Integrative Genomics Viewer (IGV V.2.1, Broad Institute, Cambridge, Massachusetts, USA), and with the support of publically available datasets reporting on their established or predicted oncogenicity (i.e. COSMIC, OncoKB and Mutation Assessor via cBioPortal). All molecular analyses were carried out in tissue samples collected before the administration of first-line chemotherapy for advanced disease.

### Statistical analysis

Descriptive statistics were computed for all the variables of interest (clinical, pathological and molecular). The relationship between categorical variables was assessed with the Pearson’s Chi squared test of independence (2-tailed) or the Fisher exact test, depending upon the size of the groups compared. The Kaplan–Meier product-limit method and the log-rank test were used for estimating and comparing survival curves. Variables potentially affecting PFS were tested in univariate Cox proportional hazard models (ECOG-PS: 0 vs 1–2; stage: locally advanced vs metastatic; localization: stomach vs junction; number of metastatic sites: 1 vs 2–3; peritoneal metastasis: no vs yes; first-line taxane-containing chemotherapy: no vs yes; γ-H2AX/pATM: negative/single positive vs double positive) [35]. The final multivariate Cox models were obtained with variables testing significant at the univariate analysis, and by adjusting for all plausible predictors of the outcome of interest (PFS). The related estimates were reported as hazard ratio (HR) and 95% confident interval (CI). The consistency of the TAZ^pos^/WNT^mut^ model was assessed through a re-sampling without replacement method (internal validation). More specifically, 100 hundred, less-powered datasets were generated by randomly removing ~ 20% from the original sample. For each simulation, the univariate Cox model was repeated and the replication rate was calculated. Level of significance was defined at p < 0.05. Statistical analyses were carried out using SPSS version 21.0 (SPSS Inc., Chicago, Illinois, USA).

## Results

### Baseline characteristics of the study participants

Baseline characteristics of the 86 patients included in the present study are summarized in Table 1. Median age at diagnosis was 61 years (IQ range 53.6–67.6). 36 (41.9%) and 50 (58.1%) patients had a locally advanced or metastatic disease, respectively. 50 (58.1%) patients received three-drug chemotherapy, and taxane-containing regimens were administered to 47 (54.7%) patients. In this series, 42 (48.8%) patients were treated within the context of prospective phase II trials. We did not record any significant association between the investigational biomarkers (YAP, TAZ, Wnt mutations) and basal clinical and pathological characteristics detailed in Table 1 (data available upon request).

**Table 1:** Baseline characteristics of gastric cancer patients included in this study (N = 86)

Characteristics	N (%)
Age at diagnosis, median (min–max) [IQ range]	61 (28–79) [53.6–67.6]
Gender	
Male	44 (51.2)
Female	42 (48.8)
ECOG PS	
0	45 (52.3)
1–2	41 (47.7)
Stage	
Locally advanced	36 (41.9)
Metastatic	50 (58.1)
Previous surgery	
No	33 (38.4)
Yes	53 (61.6)
Neoadjuvant/adjuvant chemotherapy	
No	61 (70.9)
Yes	25 (29.1)
Lauren classification	
Intestinal	35 (40.7)
Diffuse	41 (47.7)
Mixed	10 (11.6)
Grade	
G2	21 (24.4)
G3	65 (75.6)
Localization	
Esophagogastric junction	6 (7.0)
Stomach	80 (93.0)
Agents (N)	
2	36 (41.9)
3	50 (58.1)
Taxanes (first-line)	
No	39 (45.3)
Yes	47 (54.7)

### Relationship between YAP/TAZ expression and Wnt mutations

**Fig. 1:**
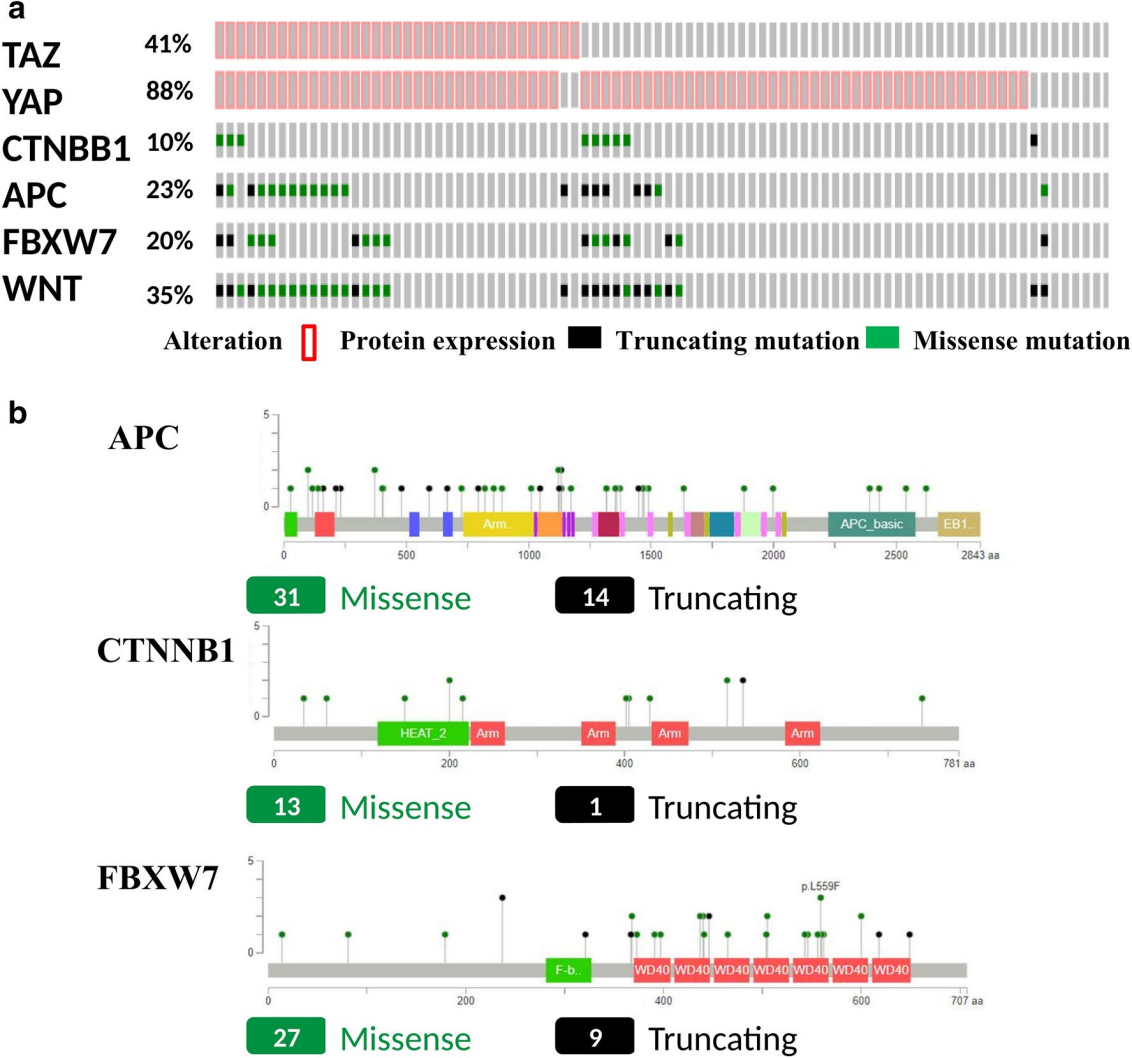
**a** Oncoprint showing the distribution of the investigated biomarkers (YAP, TAZ, *CTNNB1*, *APC*, *FBXW7*) together with the cases with at least one mutations in Wnt pathway components (integrated pathway analysis). **b** MutationMapper illustrating the entire set of detected mutations (and their nature) represented on the linear proteins

The individual distribution of the molecular biomarkers evaluated in the present study is illustrated in Fig. 1a. YAP and TAZ positivity was observed in 76 (88.4%) and 35 (40.7%) tumor samples, respectively. Mutations of *CTNNB1*, *APC* and *FBXW7* were detected in 9 (10.5%), 20 (23.2%), and 17 (19.8%) tumor samples, respectively. When considering integrated pathway analysis, 30 (34.9%) tumors carried at least one mutations in Wnt pathway genes (Fig. 1a). Overall, 95 mutations were detected: *CTNNB1* N = 14, *APC* N = 45 and *FBXW7* N = 36 (Fig. 1b). In the search of predictive factors, we first sought to address the relationship between the various investigational biomarkers. We observed a significant association between nuclear TAZ expression and the presence of Wnt mutations (Chi-squared p = 0.008) (Table 2). Prompted by this observation, we conducted an outlier analysis in the attempt of identifying molecular features that characterize exceptional responders and patients with intrinsically chemoresistant disease. Thus, we verified the distribution of YAP, TAZ, and Wnt mutations in negative and positive outliers, defined as patients in the lowest (PFS < 3.3 months) and highest (PFS ≥ 11.0 months) quartile (N = 43). When biomarkers were individually considered, only TAZ was significantly more expressed in the negative outlier group (Chi-squared p = 0.044) (Table 3). However, the strongest association was observed when we tested a signature that combined nuclear TAZ expression and Wnt mutations (TAZ^pos^/WNT^mut^), which was significantly overrepresented in the negative outlier group (Fisher p = 0.021) (Table 3). Considering the significant association between nuclear TAZ expression and Wnt pathway mutations (Table 2), results from the outlier analysis that indicate a different distribution of the TAZ^pos^/WNT^mut^ signature between negative and positive outliers (Table 3), and taking into account the biological plausibility of the TAZ^pos^/WNT^mut^ model, this molecular profile was further investigated for its impact on survival outcomes.

**Table 2:** Association between the expression of YAP/TAZ and Wnt pathway mutations (N = 86)

	WNT		p-value
	WT	MUT	
	N (%)	N (%)	
TAZ^neg^	39 (76.5)	12 (23.5)	0.008 (Chi-squared)
TAZ^pos^	17 (48.6)	18 (51.4)	
YAP^neg^	7 (70.0)	3 (30.0)	0.999 (Fisher)
YAP^pos^	49 (64.5)	27 (35.5)

**Table 3:** Association between Hippo- and Wnt-related biomarkers and positive/negative outliers (N = 43)

	PFS (outliers)		Chi-squared test
	< 1 quartile	> 3 quartile	p-value
	N (%)	N (%)	
TAZ^neg^	10 (37.0)	17 (63.0)	0.044
TAZ^pos^	11 (68.8)	5 (31.3)	
YAP^neg^	2 (40.0)	3 (60.0)	0.999*
YAP^pos^	19 (50.0)	19 (50.0)	
WNT^wt^	11 (39.3)	17 (60.7)	0.087
WNT^mut^	10 (66.7)	5 (33.3)	
Other	14 (40.0)	21 (60.0)	0.021*
TAZ^pos^/WNT^mut^	7 (87.5)	1 (12.5)	
Other	12 (41.4)	17 (58.6)	0.159
YAP^pos^/WNT^mut^	9 (64.3)	5 (35.7)	

* Fisher’s exact test

### Association between the TAZ^pos^/WNT^mut^ signature and survival outcomes

**Fig. 2:**
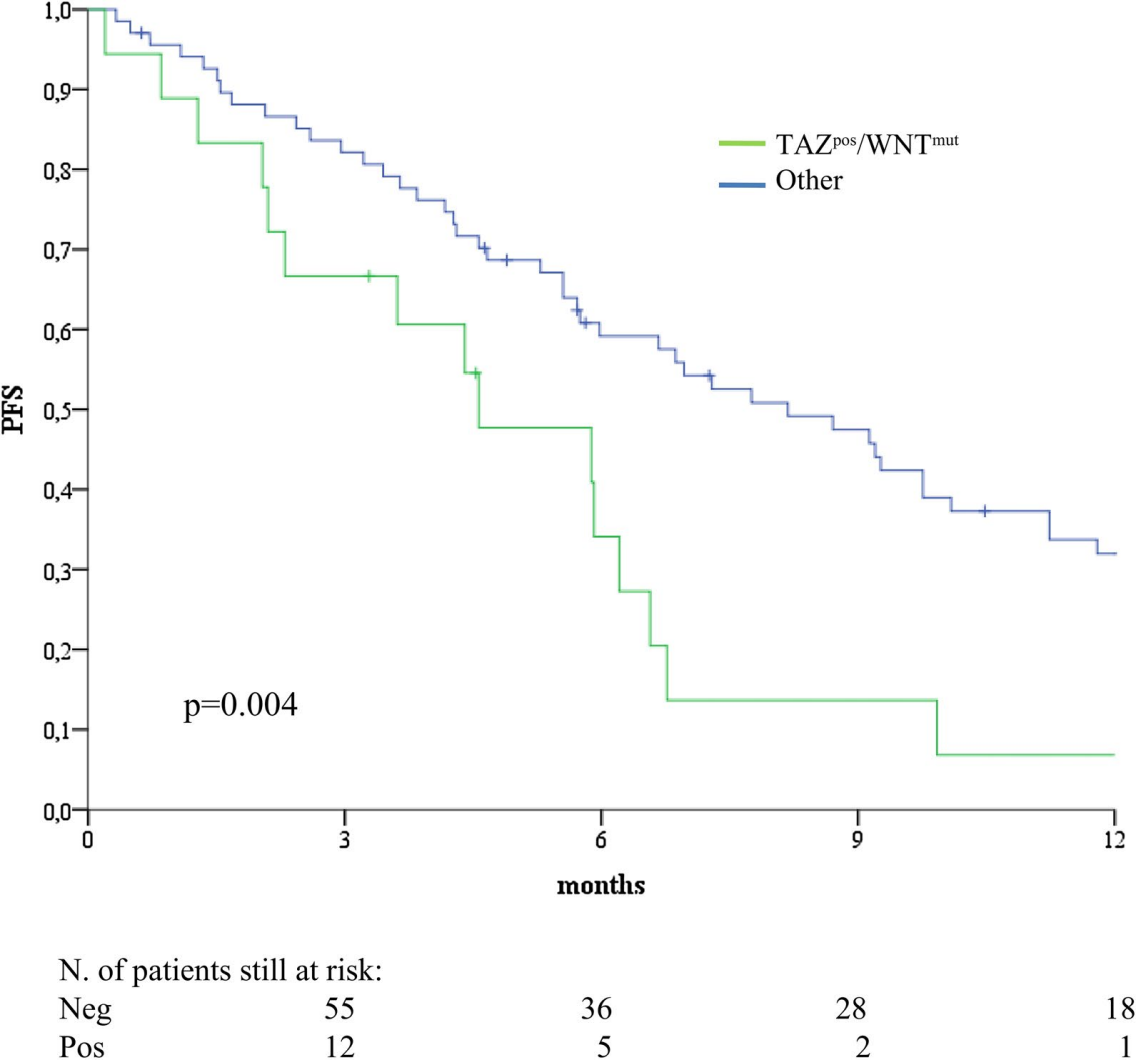
Kaplan–Meier survival curves of progression-free survival comparing TAZ^pos^/WNT^mut^ cases versus their negative counterparts (N = 86)

**Fig. 3:**
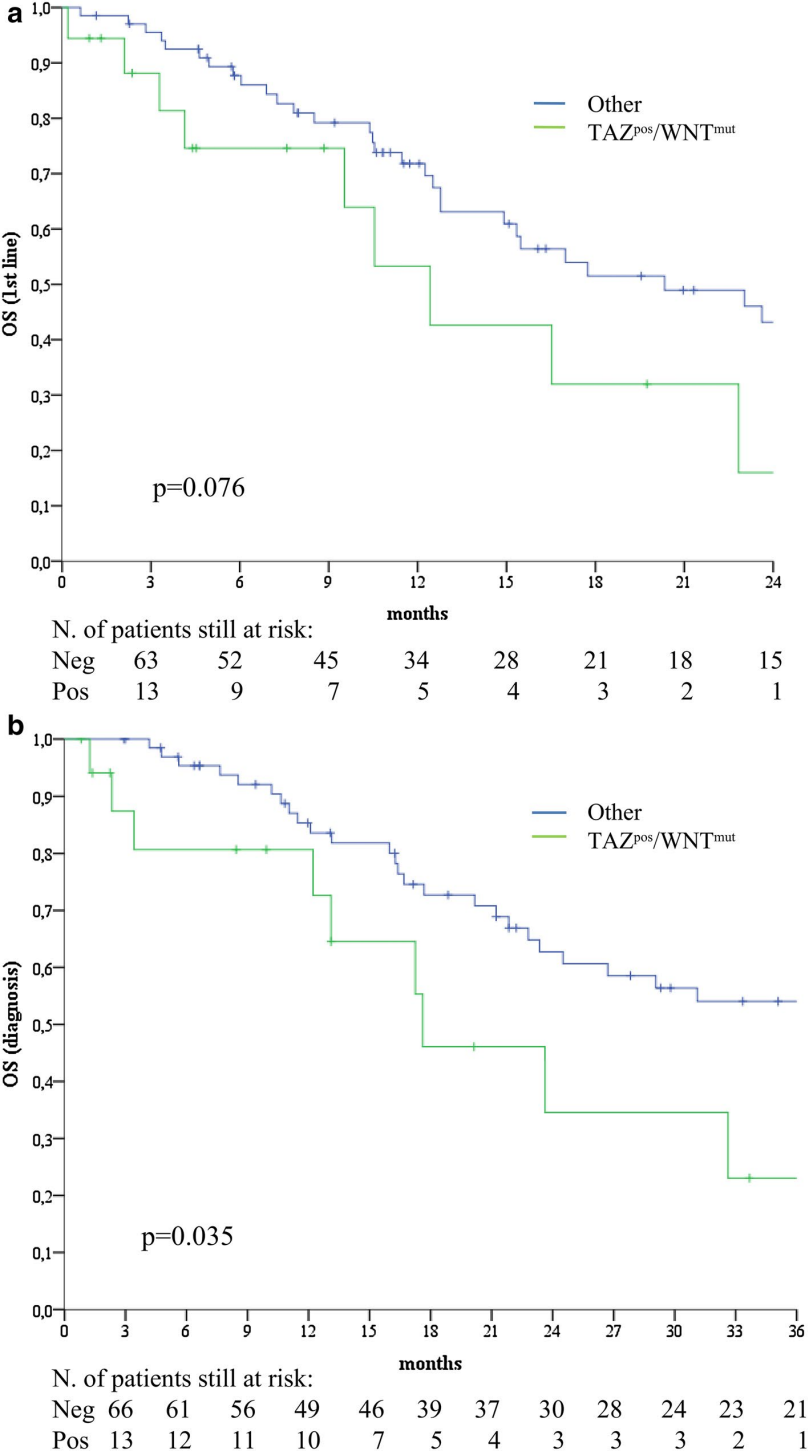
Kaplan–Meier survival curves of overall survival comparing TAZ^pos^/WNT^mut^ cases versus their negative counterparts (N = 86). **a** Refers to overall survival calculated from the first cycle of chemotherapy, whereas **b** illustrates overall survival calculated from diagnosis

Patients whose tumors carried the TAZ^pos^/WNT^mut^ signature experienced significant shorter PFS compared with their negative counterparts (log rank p = 0.004) (Fig. 2). In the univariate Cox regression analyses, the TAZ^pos^/WNT^mut^ signature was the only variable associated with an increased risk of progression (HR 2.27, 95% CI 1.27–4.05, p = 0.006) (Table 4), together with a DNA damage repair signature we previously developed in a larger cohort of 110 GC patients (the γ-H2AX^pos^/pATM^pos^ model) [35]. In the multivariate Cox models obtained by adjusting for all the plausible predictors tested in univariate analysis, the TAZ^pos^/WNT^mut^ signature remained associated with an increased risk of disease progression (HR 2.73, 95% CI 1.41–5.29, p = 0.003) (Table 4). Comparable results emerged when exclusively adjusting for the γ-H2AX^pos^/pATM^pos^ signature (HR 2.21, 95% CI 1.23–3.97, p = 0.008) (Table 4). Collectively, these data indicate that the TAZ^pos^/WNT^mut^ signature confers an increased risk of disease progression, and suggest that two independent molecular predictors were identified (TAZ^pos^/WNT^mut^ and γ-H2AX^pos^/pATM^pos^). Upon resampling (procedure detailed in “Statistical analyses” section), the replication rate for the univariate Cox model for PFS was 84%, thus indicating the stability of the model. Finally, the TAZ^pos^/WNT^mut^ signature was associated with inferior overall survival, albeit to a not fully significant extent (log rank p = 0.076) (Fig. 3a). Nevertheless, this association became fully significant when OS was computed from the time of diagnosis instead of date at the initiation of chemotherapy (log rank p = 0.035) (Fig. 3b), suggesting that the TAZ^pos^/WNT^mut^ signature may hold both predictive and prognostic significance.

**Table 4:** Uni- and multivariate Cox regression models for PFS (N = 86)

	Univariate Cox regression model		Multivariate Cox regression model^a^		Multivariate Cox regression model^b^	
	HR (95% CI)	p-value	HR (95% CI)	p-value	HR (95% CI)	p-value
γ-H2AX^pos^/pATM^pos^						
Positive vs other	2.14 (1.30–3.53)	0.003	2.09 (1.27–3.45)	0.004	1.87 (1.04–3.39)	0.038
TAZ^pos^/WNT^mut^						
Positive vs other	2.27 (1.27–4.05)	0.006	2.21 (1.23–3.97)	0.008	2.73 (1.41–5.29)	0.003
ECOG-PS						
1–2 vs 0	1.23 (0.77–1.97)	0.391			1.20 (0.73–1.95)	0.471
Stage						
Met vs loc adv	1.12 (0.69–1.80)	0.647			0.89 (0.46–1.73)	0.737
Localization						
Stomach vs EOJ	0.67 (0.27–1.69)	0.398			1.54 (0.53–4.42)	0.424
Number of metastatic sites						
2–3 vs 1	1.57 (0.93–2.65)	0.089			1.33 (0.70–2.50)	0.379
Peritoneal metastasis						
Yes vs No	0.67 (0.42–1.07)	0.097			0.72 (0.41–1.27)	0.261
Taxanes						
Yes vs No	0.93 (0.58–1.51)	0.784			0.79 (0.45–1.40)	0.429

^a^Adjusted for the variables significant at the univariate analysis

^b^Adjusted for all the variables tested at univariate analysis

## Discussion

In the present study, we examined the expression of the Hippo transducers YAP/TAZ together with mutations in central components of the Wnt pathway in a relatively large series of advanced GC patients treated with chemotherapy in the first-line setting. Approximately half of the patients examined were treated in the context of prospective phase II trials [27–30]. This study, which is hypothesis-generating by nature, capitalizes on a growing body of evidence that converge on assigning to the Hippo–Wnt pathway cooperation a central role in three intertwined processes, namely organ development, tissue repair after injuries and tumorigenesis [22, 23]. Collectively, our results indicate that: (i) a subset of GC is characterized by a signature denoting deregulation of both Hippo and Wnt, (ii) the coexistence of nuclear TAZ expression and pathogenic Wnt pathway mutations seems to be predictive of shorter PFS, and then reduced efficacy of first-line chemotherapy, and (iii) the TAZ^pos^/WNT^mut^ signature may also represent an adverse prognostic factor. To our knowledge, this is the first report striving to address the clinical significance of the Hippo–Wnt crosstalk in GC. Earlier studies suggested that YAP/TAZ are often expressed in GC, which is consistent with our data [24, 36–38]. Nevertheless, studies reported so far have described small-sized case series without a clear focus on therapeutic outcomes (e.g. by pooling data concerning patients with various disease stages and prognosis), or have been conducted in specific disease entities which are not necessarily representative for the overall category of advanced GC (e.g. signet ring cell carcinoma, gastroesophageal junction cancers) [24, 36–38].

In our opinion, our findings raised a number of points that may streamline the identification of Hippo/Wnt-related predictive factors in GC. First, the molecular characterization of GC delineated four distinct molecular subtypes: chromosomal instability (CIN), microsatellite instability (MSI), genomically stable (GS) and Epstein–Barr virus (EBV)-positive [26]. Mutations in Wnt pathway components were observed across all non-hypermutated subtypes. Conversely, hallmarks of GS–GC are *RHOA* and *CDH1* mutations, together with *CLDN18*–*ARHGAP26* fusions. All these alterations suggest genetically-driven deregulation of the Hippo pathway. Indeed, Rho GTPases are involved in the activation of YAP/TAZ and in the inhibition of Hippo kinases via two distinct mechanisms: (i) stimulation by soluble factors that act through G-protein-coupled receptors (GPCRs) and Rho GTPases [39–43], and (ii) mechanical cues, such as extracellular matrix stiffness and changes in cell geometry, attachment status and density, that regulate YAP/TAZ through Rho GTPases and the remodeling of the F-actin cytoskeleton [44–46]. Next, *CDH1* encodes for the cell–cell adhesion molecule E-cadherin, the central component of adherens junctions. E-cadherin is an established positive regulator of MST1/2 activity, whereas the E-cadherin-associated protein α-catenin sequesters YAP/TAZ in the cytoplasm, hindering their nuclear translocation [47–49]. Consistently, disruption of the E-cadherin–catenin complex at the cell–cell junction fuels YAP/TAZ activation [47–49]. Finally, the *CLDN18*–*ARHGAP26* fusion implies defects in *CLDN18* and *ARHGAP26*. *CLDN18* encodes for Claudin 18, a component of tight junctions (TJs) [50]. TJ proteins promote activation of Hippo kinases and/or sequester YAP/TAZ in the cytoplasm, whereas *ARHGAP26* encodes for the Rho-Type GTPase-Activating Protein 26 [51–57]. These observations suggest that GS–GC is characterized by multiple defects in cell–cell adhesion mechanisms that, in turn, can propel YAP/TAZ activation. Different considerations apply to EBV-related GC. Experimental models of liver and cervical tumors are beginning to shed light on the connection between viral proteins and YAP/TAZ. For instance, the hepatitis B virus X protein (HBx) up-regulates YAP promoting the growth of hepatoma cells, whereas in hepatocellular carcinoma cell lines the transcriptional activator PreS2 up-regulates TAZ via the suppression of miRNA-338-3p [58, 59]. Likewise, in cervical cancer cells the HPV E6 protein protects YAP from proteasome-dependent degradation in a process that ignites cancer cell proliferation [60]. Remarkably, a distinctive feature of EBV-associated GC is the extreme DNA hypermethylation, and both *MOB1B* and *WWTR1* (the gene encoding for TAZ) present frequent promoter hypermethylation [26]. Thus, more tailored investigations are needed in the future, which specifically take into account the molecular classification of GC and the underlying molecular portraits characterizing the different subtypes.

Another aspect that deserves mention is that the activity of Hippo and Wnt is modulated by negative feedback loops. Indeed, the YAP/TAZ–TEADs and β-catenin-TCF/LEF complexes also promote the transcription of negative pathway regulators [61, 62]. For instance, the YAP/TAZ–TEADs complex controls the activity of Hippo kinases by inducing the expression of LATS2, and mediates the transcription of neurofibromin 2 (NF2, also known as Merlin), an established positive regulator of LATS1/2 kinase activity [61]. Aware of these mechanisms, our original experimental workflow envisioned targeted RNA sequencing for evaluating two signatures denoting the activation of YAP/TAZ and Wnt. The logic behind this was to carry out an extensive characterization at three different levels (protein, transcript and gene), which would have enabled us to investigate negative feedback loops. Even though this task was halted owing to excessive RNA degradation in the majority of samples, transcript-level analysis will be further pursued in future studies from our research team.

Finally, Hippo and Wnt are two pieces of a wider cross-regulation process involving multiple signaling pathways [i.e. Hedgehog, Notch and Bone Morphogenetic Protein (BMP)], whose activity is central in organ development, tissues homeostasis, stem cell fate and tumorigenesis [19, 20]. Albeit these pathways are not targeted by genetic events in GC [26], the evaluation of biomarkers functioning as readout for their activation may add further granularity, allowing the evaluation of co-regulated signaling avenues.

## Conclusions

Our data pointed to the combined activation of two oncogenic avenues, YAP/TAZ and Wnt, as potential biomarkers for predicting the efficacy of chemotherapy in GC patients. Considering the intricate molecular network that co-regulates YAP/TAZ and Wnt, we believe that more comprehensive, subtype-restricted, pathway analyses will be instrumental to gain a better understanding on the Hippo–Wnt pathway crosstalk and its clinical implications.

## Additional files

**Additional file 1.** First-line chemotherapy regimens and schedules (N = 86).

**Additional file 2.** Representative examples of immunohistochemical expression of TAZ and YAP in gastric cancer. Two cases are presented with combined nuclear expression of both TAZ and YAP (A–D).

## Abbreviations

APC: Adenomatosis Polyposis Coli
CTNNB1: β-catenin
FBXW7: F-box/WD repeat domain-containing 7
GC: gastric cancer
GPCRs: G-protein-coupled receptors
LATS1 and LATS2: large tumor suppressor 1 and 2
MOB1A and MOB1B: MOB kinase activator 1A and 1B
MST1 and MST2: sterile 20-like kinase 1 and 2
OS: overall survival
PFS: progression-free survival
SAV1: Salvador homolog 1
TAZ: transcriptional co-activator with PDZ-binding motif
TEAD1-4: TEA domain-containing sequence-specific transcription factors
YAP: yes-associated protein
