# Supplementary material for: Expression of the Hippo transducer TAZ in association with WNT pathway mutations impacts survival outcomes in advanced gastric cancer patients treated with first-line chemotherapy
Source: J Transl Med. 2018 Feb 5;16:22. doi: 10.1186/s12967-018-1385-y (PMC5800016; doi:10.1186/s12967-018-1385-y)
Supplement: Supplementary file 1 — Additional file 1. First-line chemotherapy regimens and schedules (N = 86). [file 12967_2018_1385_MOESM1_ESM.doc]

Additional file 1: First-line chemotherapy regimens and schedules (N=86).

| **Regimen** | **Schedule** | **N°** |
| --- | --- | --- |
| SALTZ | Irinotecan 125 mg/m2, AF 20mg/m2 and 5-FU 500 mg/m2 D1, 8, 15 and 21, q42 days | 9 |
| EOD* | Epirubicin 50 mg/m2 and docetaxel 60 mg/m2 followed by oxaliplatin 100 mg/m2 D1, q21 days | 8 |
| FEP | 5-FU 500 mg/m2, epirubicin 20 mg/m2 and cisplatin 20 mg/m2 D1-3, q28 days | 5 |
| CF (+/- trastuzumab)** | Cisplatin 80 mg/m2 D1, 5-FU 800 mg/m2 22-h continuous infusion D1-4, trastuzumab 6 mg/Kg D1 (8 mg/kg loading dose), q21 days | 3 |
| DCF | 5-FU 1000 mg/m2 22-h continuous infusion D1-4, docetaxel 75 mg/m2 and cisplatin 75 mg/m2 D1, q21 days | 2 |
| DDP-TXT | Docetaxel 75 mg/m2 and cisplatin 75 mg/m2 D1, q21 days | 3 |
| D-Folfox* | Docetaxel 60 mg/m2 and oxaliplatin 100 mg/m2 D1, AF 100 mg/m2 D1 and 2, 5-FU 400 mg/m2 bolus D1 and 2, 5-FU 600 mg/m2 22-h continuous infusion D1-2, q21 days | 4 |
| ECD* | Epirubicin 50 mg/m2 and docetaxel 60 mg/m2 D1; cisplatin 60 mg/m2 D2, q21 | 16 |
| ELF | 5-FU 500 mg/m2, AF 150 mg/m2 and etoposide 120 mg/m2 D1-3, q21 days | 5 |
| Folfiri | Irinotecan 180 mg/m2 D1, AF 100 mg/m2 D1 and 2, 5-FU 400 mg/m2 bolus D1 and 2, 5-FU 600 mg/m2 22-h continuous infusion D1-2, q14 days | 7 |
| Folfox | Oxaliplatin 85 mg/m2 D1, AF 100 mg/m2 D1 and 2, 5-FU 400 mg/m2 bolus D1 and 2, 5-FU 600 mg/m2 22-h continuous infusion D1-2, q14 days | 9 |
| IDO* | Irinotecan 150 mg/m2 and docetaxel 60 mg/m2 D1, oxaliplatin 85 mg/m2 D2, q21 | 5 |
| FUFA | AF 20 mg/m2 and 5-FU 450 mg/m2 D 1, 8 and 15, q28 days | 1 |
| DOC* | Docetaxel 60 mg/m2 and oxaliplatin 100 mg/m2 D1, capecitabine 500 mg/m2 orally twice daily continuously, q21 days | 9 |

* Patients enrolled in clinical trials; **One patient received trastuzumab.

.
