# Supplementary material for: Expression of the Hippo transducer TAZ in association with WNT pathway mutations impacts survival outcomes in advanced gastric cancer patients treated with first-line chemotherapy
Source: J Transl Med. 2018 Feb 5;16:22. doi: 10.1186/s12967-018-1385-y (PMC5800016; doi:10.1186/s12967-018-1385-y)
Supplement: Supplementary file 2 — Additional file 2. Representative examples of immunohistochemical expression of TAZ and YAP in gastric cancer. Two cases are presented with combined nuclear expression of both TAZ and YAP (A–D). [file 12967_2018_1385_MOESM2_ESM.docx]

**Additional file 2:** Representative examples of immunohistochemical expression of TAZ and YAP in gastric cancer. Two cases are presented with combined nuclear expression of both TAZ and YAP (A-B and C-D).
